# A broad scope knowledge based model for optimization of VMAT in esophageal cancer: validation and assessment of plan quality among different treatment centers

**DOI:** 10.1186/s13014-015-0530-5

**Published:** 2015-10-31

**Authors:** Antonella Fogliata, Giorgia Nicolini, Alessandro Clivio, Eugenio Vanetti, Sarbani Laksar, Angelo Tozzi, Marta Scorsetti, Luca Cozzi

**Affiliations:** Oncology Institute of Southern Switzerland, Bellinzona, Switzerland; Radiotherapy and Radiosurgery Department, Humanitas Clinical and Research Center, Milan-Rozzano, Italy; Radiotherapy Department, Tata Memorial Hospital, Mumbai, India

**Keywords:** Knowledge based planning, RapidPlan, RapidArc, Esophageal cancer

## Abstract

**Background:**

To evaluate the performance of a broad scope model-based optimisation process for volumetric modulated arc therapy applied to esophageal cancer.

**Methods and materials:**

A set of 70 previously treated patients in two different institutions, were selected to train a model for the prediction of dose-volume constraints. The model was built with a broad-scope purpose, aiming to be effective for different dose prescriptions and tumour localisations. It was validated on three groups of patients from the same institution and from another clinic not providing patients for the training phase. Comparison of the automated plans was done against reference cases given by the clinically accepted plans.

**Results:**

Quantitative improvements (statistically significant for the majority of the analysed dose-volume parameters) were observed between the benchmark and the test plans. Of 624 dose-volume objectives assessed for plan evaluation, in 21 cases (3.3 %) the reference plans failed to respect the constraints while the model-based plans succeeded. Only in 3 cases (<0.5 %) the reference plans passed the criteria while the model-based failed. In 5.3 % of the cases both groups of plans failed and in the remaining cases both passed the tests.

**Conclusions:**

Plans were optimised using a broad scope knowledge-based model to determine the dose-volume constraints. The results showed dosimetric improvements when compared to the benchmark data. Particularly the plans optimised for patients from the third centre, not participating to the training, resulted in superior quality. The data suggests that the new engine is reliable and could encourage its application to clinical practice.

**Electronic supplementary material:**

The online version of this article (doi:10.1186/s13014-015-0530-5) contains supplementary material, which is available to authorized users.

## Background

In the recent past, knowledge-based approaches were studied and applied to some components of the radiotherapy chain, in particular to treatment planning. Early pre-clinical and clinical experiments have been performed [1–7] to demonstrate the feasibility of predicting appropriate dose-volume constraints starting from appropriate modelling of historical data. The groups of the Duke University [1–5] and of the Washington University [6, 7], pioneers in knowledge-based planning, provided evidence about the improved plan quality, reduced inter-clinician variability, and about the possibility to transfer the planning expertise from more experienced centres to less experienced institutions.

In this frame, a recent commercial implementation of knowledge-based planning was released by Varian Medical Systems (Palo Alto, USA), the RapidPlan system. Early pre-clinical validation studies have been published [8–10] investigating its role for the planning of liver, prostate and lung and head and neck cancer, using VMAT and IMRT technologies. In all these studies, the primary focus was the appraisal of the quality of the models built from relatively limited sets of patients, and the determination of efficient methods for the model validation. The aim of the RapidPlan system is to enable the automatic generation of individualised dose-volume constraints for any new patient based on the knowledge and the modelling of historical (or library-based) planning data. The dose-volume constraints obtained from this modelling should result: i) consistent with the consolidated clinical practice, ii) achievable, i.e. encompassing and solving possible trade-offs between conflicting clinical objectives and iii) optimal, in the sense that the overall solution should represent the best balance between all requests and the most effective healthy tissue protection, specific for the new patient. All the above can be realised in the assumption that the models are built from libraries of properly selected cases, i.e. cases optimised by experienced planners with the implementation of state-of-the-art clinical objectives. The use of this approach might streamline the optimisation process by minimising the need of interactive or iterative determination of planning objectives and, also, increase the consistency and the transferability of planning knowledge within and among a network of institutions.

Volumetric modulated arc therapy (VMAT) has been investigated for esophageal cancer in a limited number of planning studies [11–18], and clinical results are still to be provided. From these investigations, VMAT resulted technically feasible with superior results in terms of dose distributions (target coverage and organs at risk (OAR) sparing) compared to 3D conformal therapy and also to fixed field intensity modulated therapy (IMRT).

The aim of the present study was to demonstrate the possibility to build, using a commercial system, a predictive model able to generate dose volume histogram and constraints for optimizing VMAT plans for esophageal cancer patients. Plan data from the databases of three institutions were selected for the study. Data from two clinics, with similar patient recruitment and planning strategies were used for the model definition and training. Independent groups of plans from all three clinics, not used for the training, were then used for the model validation. The rationale for keeping clinic C in the validation phase only, is to understand if a model generated from plans with certain characteristics can be effectively used in a broad-scope to transfer expertise from more experienced centres to either less experienced or more peripheral institutions with less resources and/or excessive workload.

## Matherial and methods

A new knowledge-based optimisation engine, named RapidPlan, was introduced in the Eclipse treatment planning system (Varian Medical Systems, Palo Alto, USA) from its release 13.5. First studies on pre-clinical validation have been recently published [8–10] and details on its implementation in Eclipse and its main features can be therein found. A more detailed description of the mathematical implementation and of the algorithms applied can be found in [19, 20]. In summary, RapidPlan has three components: i) a model building and training environment (DVH Estimation Model Configuration); ii) an automated model based dose-volume constraints prediction tool (DVH Estimation); iii) a new VMAT and IMRT optimisation algorithm (PO, Photon Optimizer).

### The DVH estimation model configuration

A model uses a set of plan optimisation rules (chosen objectives and priorities) for structures (target volumes or OARs) included in the model itself. Objectives and priorities can be manually and numerically assigned, or generated for the specific patient by the model. The model is configured from a number of relevant geometric and dosimetric features from a set of selected plans. During the configuration process, a combination of Principal Component Analysis and regression techniques (PCA-regression) is applied for the in-field region of the OARs, and a mean and standard deviation model for the other OAR regions. The final result is a set of model parameters that are used in the next step to estimate the DVHs for a new case.

### Built-in model training evaluation

A statistical summary about the goodness of the model is produced as an output of the training phase. Some parameters provide assessment about the model goodness-of-fit and will be summarised in the results. The DVH’s and GED’s (Geometry-based Expected Dose) principal component average fits indicate the percentage of cases in the training properly reconstructed by the model. The coefficient of determination of the regression model parameters and the whole estimation model fit, measure the variance and the quality of the regression model: a good fit gives value close to 1. The average chi square of the regression model parameters (Pearson’s chi-squared test) represents the difference between the original and the estimated data: the closer to 1 the better the fit. The goodness-of-estimation is expressed by the mean squared error between original and estimate, that measures the distance between the original DVH and the mean of the upper and lower bounds of the estimated DVH: the closer to 0 the better. The percentage of dose bins falling outside the estimation bounds should be 32 % in the ideal case (out of 1 SD).

### The DVH estimation

This component is used for generating estimated DVH and optimization objectives for a plan of a new patient. From the DVH estimation model parameters, the most probable, upper and lower bound DVHs are generated using the PCA-regression model for the OAR in-field region, and the mean and standard deviation model for the other OAR regions. Once the upper and lower bound DVHs are computed, the objective generation phase determines the dose volume constraints (lines and/or points, user definable) to use in the optimization process, according to the choices in the model configuration. Users might also be free to add further objectives, modify priorities and perform interactive optimisation if needed.

### Model definition and validation

*Patients selection:* the case of esophageal cancer was selected for this investigation.

Seventy patients, from two clinics, previously planned with RapidArc VMAT (Varian, Palo Alto, USA) were selected for the training. All plans were approved for clinical use. Forty patients were provided by clinic A with dose prescriptions ranging from 40 to 60Gy (1.8-2.5 Gy/fraction); 30 patients were selected from the database of clinic B and were all planned with a dose prescription of 41.4Gy (1.8Gy/fraction). Patients were select to sample with equal proportions the three districts for the localisation of the PTV (upper, medial and lower third of the esophagus). No other special criteria were applied for the selection of training cases except the fact that were all judged clinically acceptable and usable for treatment.

Additional 40 patients, not used for the model configuration and training, were selected for the validation; 15 each from clinics A and B and 10 from a third clinic C (this was added to better test the generalisation power of the system). The latter group was characterised by a different strategy in target definition, and a dose prescription of 50.4Gy (1.8Gy/fraction). Clinic C is experienced in the RapidArc technique, but has an extreme workload and relatively limited resources compared to the others. Details about the planning strategy of the group C can be found in [13].

The clinical target volume (CTV) was delineated to include the gross tumour and nodal disease, as identified from the endoscopic and the imaging studies. Regional nodes and the celiac axis were also added to the CTV. The CTV was created by the expansion of CTV with 20 mm in the craniocaudal and 15 mm in the radial directions. The planning target volume was obtained with an uniform expansion of 5mm from the CTV.

*DVH estimation model characteristics:* the model was here configured to give line-type objectives for all involved OARs with optimisation priorities generated by the system. A line-type objective is in theory defined as a continuous objective line representing the desired DVH; objectives of this type would maximize the DVH constraint strength in the whole dose range. In practice, continuous lines are represented by a discrete number of dose-volume constraint points and in the Eclipse implementation these are at least 5 equally spaced over the dose range of the DVH. The usage of generated priorities would leave the model to find the best possible solution for the specific patient and related anatomy; fixed priorities are more for a template-based approach. Constraints and priorities for the PTV and CTV volumes were manually set to predefined values [9] to ensure coverage.

The OARs included in the study were: left and right lungs, heart and spinal cord (spine). For patients with target in the mid or lower-third of the esophagus, additional OARs were included: liver, spleen, left and right kidneys, stomach and small bowel. The average PTV volume was 650 ± 270cm^3^ (range: 125-1209cm^3^) for the training dataset and 397 ± 250cm^3^ (range: 54-1097cm^3^) for the entire validation cases.

The Acuros-XB dose calculation algorithm was adopted with a dose resolution of 2.5mm. Acuros was applied as the algorithm for the final dose calculation as well as for the so-called intermediate dose calculation. In this study the intermediate dose calculation was automatically run at the end of the optimisation phase to refine the convergence to the planning objectives and it results particularly effective when air cavities or significant tissue heterogeneities, especially with low densities, are involved. Plans were optimised for 6MV photon beams for RapidArc with one or two full arcs. All plans were normalised to the mean dose to PTV. Standard DVH analysis was performed to appraise the quality of the model-based optimised plans versus the clinically accepted baseline benchmark. Normal distribution of data was assessed and confirmed.

A number of dose-volume objectives were used to appraise the quality of the reference and RapidPlan dose distributions and were quantitatively analysed for PTV and OAR. All objectives are listed in detail in the result tables. As reference, the clinically accepted plans were selected.

To further appraise the RapidPlan data in comparison with the reference plans, for each patient, PTV and OAR and for all dose volume objectives (a total of 564 data points) a pass-fail analysis was performed. Data were grouped in 4 classes: class 1 for failed-passed cases (reference-failed and RapidPlan-passed); class 2 for passed-failed; class 3 for failed-failed and class 4 for passed-passed. A test-point is defined as passed if the value in the plan (either RapidPlan of the reference) improved the dose-volume objective and failed in the opposite case.

## Results

A qualitative and quantitative overview of the output of the model configuration phase can be found in the Additional file 1. Table 1 reports a summary of the model training statistics. Some structures were present only in the cases where the PTV was located in the mid and mid-lower thirds of the esophagus. The quality of the regression appeared good: more than 99 % of the cases were reproduced in the DVH or GED (97 % for the left kidney) components, with an average chi square of 1.08 ± 0.04.

**Table 1:** Summary the model training statistics

	Lung left	Lung right	Heart	Spine	Liver
Structures in model	70	70	70	70	65
Estimation model goodness of fit					
DVH average fit	0.99	0.99	0.99	0.99	0.99
GED average fit	0.99	0.99	0.99	0.99	0.99
Coeff. of determination	0.83	0.92	0.73	0.49	0.88
Whole estimation model fit	0.83	0.91	0.73	0.49	0.87
Average chi square	1.07	1.09	1.04	1.05	1.05
Model goodness of estimation					
MSE original and estimate	0.001	0.001	0.005	0.01	0.002
Dose bins outside bound.[%]	41	42	35	28	41
	Spleen	Stomach	Left kidney	Right kidney	Small bowel
Structures in model	60	64	34	31	40
Estimation model goodness of fit					
DVH average fit	0.99	0.99	0.99	0.99	0.99
GED average fit	0.99	0.99	0.97	0.99	0.99
Coeff. of determination	0.88	0.95	0.79	0.84	0.95
Whole estimation model fit	0.87	0.95	0.76	0.82	0.94
Average chi square	1.02	1.09	1.08	1.11	1.15
Model goodness of estimation					
MSE original and estimate	25	0.002	0.01	0.004	0.003
Dose bins outside bound.[%]	0.005	39	29	23	44

**Fig. 1:**
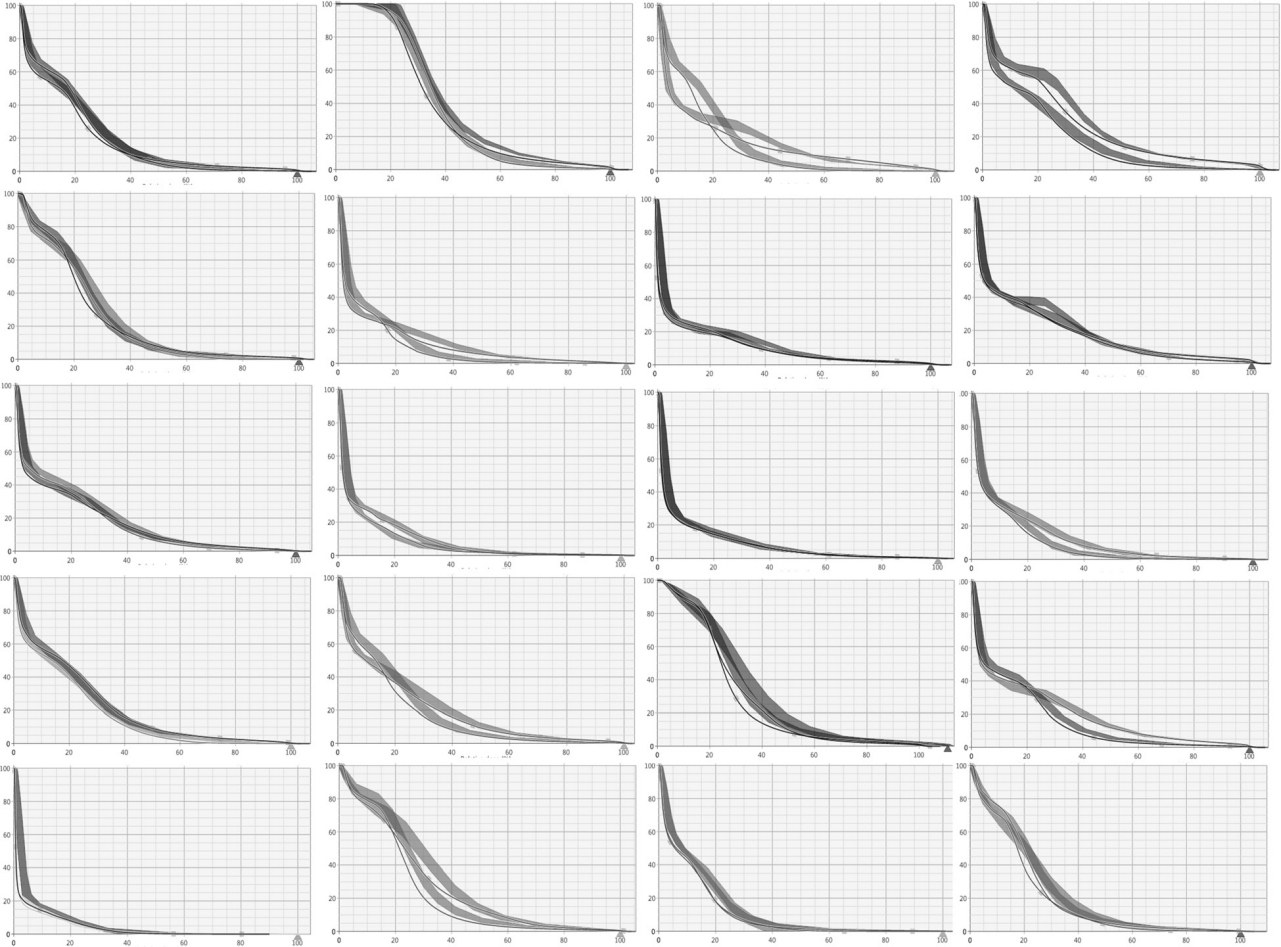
Examples of estimated DVH ranges (solid bands) and final DVH after optimisation with the model-based algorithm. Data are shown for left and right lungs for 20 of the 40 patients (chosen every second patient) used for the validation. Similar patterns for the other patients and organs at risk

Figure 1 shows, for 20 of the 40 cases (chosen every second patient, i.e. almost randomly since patients were not ordered according to any relevant parameter like tumor volume or localisation) used in the validation (including all the 10 cases from center C), the prediction bands generated by the DVH estimation engine as well as the final DVH lines after full optimisation and calculation for the left and right lungs. To notice: i) the estimation bands are narrow, ii) the final DVH falls normally below the lower limit of the bands (according to the generated line objective). Both observations support the good prediction power of the model.

**Fig. 2:**
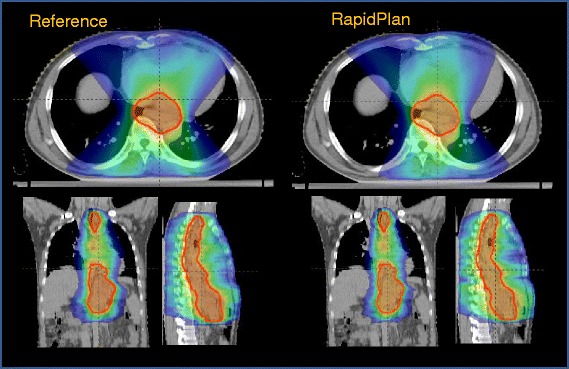
Example dose distributions for the reference plan and for the model-based optimised plan for the clinic C. The colour-wash is from 20 % to 110 % of the prescription dose (50.4Gy)

**Fig. 3:**
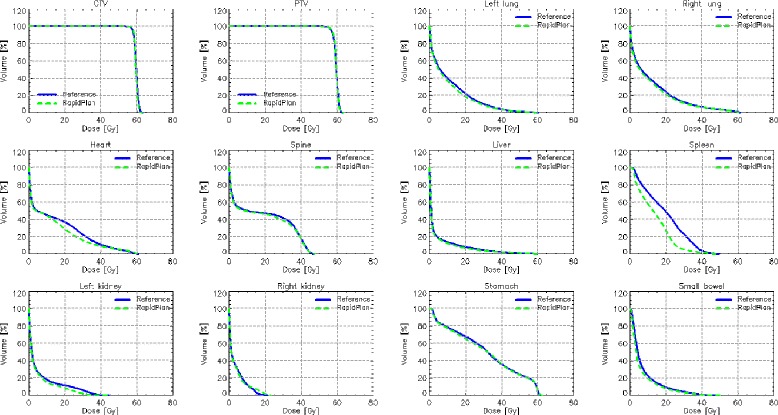
Average DVH for target volumes and organs at risk for the validation experiment for the clinic C. The Reference lines are for the original plans manually optimised, while the RapidPlan lines are for the model-based optimisation

Figure 2 shows the dose distributions (the colorwash is in the range 20–110 %) for one case from clinic C. The comparison is between the reference plan (left panels) and the RapidPlan data (right panels). Figure 3 reports the average DVHs for CTV, PTV and OAR comparing the reference and the RapidPlan data for clinic C (Additional file 1: Figures S4 and S5 show the same for the clinics A and B). RapidPlan allowed to modestly improve some of the OARs (e.g. heart) with some more remarkable effect on the spleen.

Table 2 presents the summary of the quantitative analysis of the DVH for the entire group of 40 patients in the validation; p values are reported only when significant (<0.05) or when a tendency to significance (*p* < 0.1) was observed. The data confirm that modest but systematic improvements can be achieved with RapidPlan. Table 3 presents the same summary but limited to the 10 cases from clinic C. The data resulted consistent with the general analysis despite the different strategies in target definition and OARs contouring, the variations in tumour stages, and in the dose prescriptions. On average, planning objectives were met for both the entire cohort as well as for the clinic C dataset.

**Table 2:** Summary the DVH analysis for the reference and the RapidPlan plans for the entire cohort of validation patients from the 3 clinics

	Objective	Reference	RapidPlan	*p*
PTV				
Mean [%]	100 %	100.0 ± 0.0	100.0 ± 0.0	-
D_1%_ [%]	<107 %	104.2 ± 3.2	103.8 ± 3.1	-
V_95%_ [%]	>95 %	97.3 ± 2.9	98.0 ± 1.2	-
St. Dev. [%]	<5 %	2.1 ± 0.1	2.0 ± 0.1	-
Left lung				
Mean [Gy]	<15Gy	8.9 ± 4.2	9.1 ± 4.8	-
V_20Gy_ [%]	<20%	12.4 ± 10.2	12.6 ± 11.0	-
Right lung				
Mean [Gy]	<15Gy	9.2 ± 4.8	9.1 ± 5.2	-
V_20Gy_ [%]	<20 %	12.1 ± 11.3	12.2 ± 11.7	-
Heart				
Mean [Gy]	<25Gy	11.9 ± 9.9	10.7 ± 8.3	0.03
V_30Gy_ [%]	<30 %	10.3 ± 16.1	7.0 ± 8.9	0.07
Spine				
D_1cm3_ [Gy]	<45Gy	26.2 ± 11.5	23.6 ± 9.7	0.001
Liver				
Mean	<15Gy	8.5 ± 5.9	8.6 ± 6.4	-
Spleen				
Mean [Gy]	<20Gy	14.3 ± 7.6	12.3 ± 6.3	0.004
D_1%_ [Gy]	<40Gy	32.6 ± 10.6	30.6 ± 11.0	-
Left kidney				
V_15Gy_ [%]	<35 %	16.4 ± 16.9	12.7 ± 18.7	0.03
Right kidney				
V_15Gy_ [%]	<35 %	4.7 ± 7.3	3.4 ± 5.0	-
Stomach				
D_1%_ [Gy]	<50Gy	43.3 ± 8.8	42.5 ± 9.9	0.07
Small bowel				
Mean [Gy]	<10Gy	6.6 ± 3.1	6.3 ± 3.0	0.03
D_1%_ [Gy]	<45Gy	25.6 ± 9.2	24.6 ± 8.6	-

**Table 3:** Summary the DVH analysis for the reference and the RapidPlan plans for the cohort of validation patients from the clinic C

	Objective	Reference	RapidPlan	*p*
PTV				
Mean [%]	100 %	100.0 ± 0.0	100.0 ± 0.0	-
D_1%_ [%]	<107 %	105.0 ± 1.0	104.8 ± 0.9	-
V_95%_ [%]	>95 %	96.5 ± 2.5	97.1 ± 1.1	-
St. Dev. [Gy]	<5 %	2.5 ± 0.8	2.4 ± 0.8	-
Left lung				
Mean [Gy]	<15Gy	11.4 ± 4.7	10.6 ± 4.2	0.01
V_20Gy_ [%]	<20 %	22.4 ± 10.6	18.9 ± 7.2	0.01
Right lung				
Mean [Gy]	<15Gy	12.6 ± 5.5	11.9 ± 5.3	0.01
V_20Gy_ [%]	<20 %	24.4 ± 12.3	21.7 ± 11.2	-
Heart				
Mean [Gy]	Minimise	15.4 ± 15.1	11.9 ± 11.9	0.06
V_30Gy_ [%]	<30 %	22.7 ± 25.5	16.2 ± 18.7	0.02
Spine				
D_1cm3_ [Gy]	<45Gy	43.3 ± 3.8	43.1 ± 4.4	0.01
Liver				
Mean	Minimise	4.9 ± 7.1	4.5 ± 6.4	0.01
Spleen				
Mean [Gy]	Minimise	19.7 ± 4.2	13.9 ± 3.5	-
D_1%_ [Gy]	Minimise	45.5 ± 3.2	43.8 ± 4.1	-
Left kidney				
V_15Gy_ [%]	<35 %	14.0 ± 16.5	11.5 ± 14.3	-
Right kidney				
V_15Gy_ [%]	<35 %	3.8 ± 4.6	3.7 ± 4.9	-
Stomach				
D_1%_ [Gy]	Minimise	60.8 ± 0.6	61.5 ± 0.1	-
Small bowel				
Mean [Gy]	Minimise	7.7 ± 2.4	6.7 ± 2.3	0.01
D_1%_ [Gy]	<45Gy	39.3 ± 7.0	38.8 ± 6.9	-

Finally, Table 4 presents the results of the case-by-case pass-fail analysis conducted on all the 40 validation patients and for all the planning objectives. Of the 624 dose-volume test points, 21 (3.3 %) were scored as failure for the reference plans and pass for the RapidPlan. Of these, 14 were from the clinic C dataset. Only 3 points (<0.5 %) resulted with RapidPlan failing when the reference plan succeeded (none from group C). Overall, RapidPlan resulted equivalent or superior to the reference plans in almost the totality of the cases.

**Table 4:** Summary of the case-by-case pass-fail analysis for the selected dose-volume planning objectives for the reference and for the model-based plans for all the validation cases

	Objective	Reference fail	Reference pass	Reference fail	Reference pass
		Rapidplan pass	RapidPlan fail	RapidPlan fail	RapidPlan pass
PTV (40 cases)					
D_1%_ [%]	<107 %	0 (0 %)	0 (0 %)	0 (0 %)	40 (100 %)
V_95%_ [%]	>95 %	5 (12 %)	0 (0 %)	0 (0 %)	35 (88 %)
St. Dev. [%]	<5 %	0 (0 %)	0 (0 %)	0 (0 %)	40 (100 %)
Left lung (40 cases)					
Mean [Gy]	<15Gy	1 (2 %)	0 (0 %)	4 (10 %)	35 (90 %)
V_20Gy_ [%]	<20 %	1 (2 %)	0 (0 %)	5 (12 %)	34 (86 %)
Right lung (40 cases)					
Mean [Gy]	<15Gy	0 (0 %)	2 (5 %)	2 (5 %)	36 (90 %)
V_20Gy_ [%]	<20 %	2 (5 %)	0 (0 %)	5 (12 %)	33 (83 %)
Heart (40 cases)					
Mean [Gy]	<25Gy	2 (5 %)	0 (0 %)	2 (5 %)	36 (90 %)
V_30Gy_ [%]	<30 %	2 (5 %)	0 (0 %)	1 (2 %)	37 (94 %)
Spine (40 cases)					
D_1cm3_ [Gy]	<45Gy	3 (7 %)	0 (0 %)	1 (2 %)	36 (91 %)
Liver (31 cases)					
Mean	<15Gy	1 (3 %)	1 (3 %)	3 (10 %)	26 (84 %)
Spleen (25 cases)					
Mean [Gy]	<20Gy	1 (4 %)	0 (0 %)	1 (4 %)	23 (92 %)
D_1%_ [Gy]	<40Gy	1 (4 %)	0 (0 %)	5 (20 %)	19 (76 %)
Left kidney (16 cases)					
V_15Gy_ [%]	<35 %	0 (0 %)	0 (0 %)	1 (6 %)	15 (94 %)
Right kidney (16 cases)					
V_15Gy_ [%]	<35 %	2 (12 %)	0 (0 %)	0 (0 %)	14 (88 %)
Stomach (31 cases)					
D_1%_ [Gy]	<50Gy	0 (0 %)	0 (0 %)	2 (6 %)	29 (94 %)
Small bowel (20 cases)					
Mean [Gy]	<10Gy	0 (0 %)	0 (0 %)	1 (5 %)	19 (95 %)
D_1%_ [Gy]	<45Gy	0 (0 %)	0 (0 %)	0 (0 %)	20 (100 %)

Planning time was not part of the study design since its evaluation is prone to a number of subjective or external factors not easy to objectively quantify. In particular, the experience of individual planners and workload as well as computer hardware have a strong influence. Limiting to the RapidPlan aspects, some data were collected. Once plans are identified as good candidates for the model training, the time needed to “extract” the data and load them into the configuration workspace is limited to about 15–20 s per plan. The time needed to train a model is approximately 2 min. Being an investigational study, the time needed to validate a model prior to the dosimetric experiments, cannot be assessed and will become clearer when the learning phase will be completed. Plan optimisation with the DVH estimation engine requires about 15–20 s for the constraints generation and, in the case of esophagus, about 10–15 min of free-run optimisation inclusiding of the so-called intermediate dose calculation phase. The time needed for final dose calculation is independent from the knowledge-based or conventional approach applied for optimisation and depends on the algorithm and the case complexity. For esophageal cancer it takes about 6–8 min to perform a full dose calculation with Acuros-XB.

## Discussion

The scope of the present study was to appraise the possibility to use a knowledge-based dose-constraint prediction engine for plan optimisation of VMAT with clinically acceptable results. Previous studies [8--10] demonstrated the possibility of developing and using models for liver, lung, prostate and head and neck cancer patients. Validation tests showed that plan quality was not inferior, while in some instances superior when knowledge-based methods were applied to generate the optimisation dose-volume constraints compared to the routine clinical practice. All the published validation experiments of RapidPlan were performed using only cases pooled from the databases of the same institutions providing the model training cases. The approach of RapidPlan is based on the automated prediction of individualized dose-volume objectives from a population-based or library-based historical knowledge. This differs from other possible approaches that are currently available in clinical routine as class solutions or fixed templates or that have been presented and are investigated like the use of or selection from pools of Pareto-optimal groups of plans. The use of class solutions and templates, although simple to implement, has the limit given by the static nature of those objects. Any individualisation of the objectives is prone to the need of trial-and-error planning and might lead to sub-optimal planning when challenging patients or un-experienced planners are involved. The other type of approach is more interesting and in principle, following different paths, and should aim to the same results: an individualised determination of the best plan from the optimisation of the trade-offs between competing objectives. Several studies [21–26] investigated the role of multi-criteria optimization (a form of Pareto-optimal navigation approach) for both IMRT and VMAT and demonstrated the possibility to maximise the sparing of organs at risk with preservation of target coverage. The difference between the RapidPlan and the Pareto-optimal approaches is mostly in the methodology: the first aims to determine automatically the best personalized constraints, given the prescription requirements, the clinical objectives and the historical knowledge; the latter starts from static clinical objectives and navigates through the realm of possible solutions to find the optimal plan. The degree of automation of the process can be different between the two approaches and dedicated comparative studies would be needed to ascertain if one approach might be preferable. At the present status this evidence is missing and both methods appear to be very promising.

It is not directly relevant for the scope of these investigations to discriminate if the use of physical or biological (like EUD or NTCP) constraints are used to generate the ideal optimisation results. Incidentally, within RapidPlan, but not investigated here, it is possible to design the model with any combination of physical dose-volume constraints and/or generalised EUD for targets and OARs.

Other automated objective definition methods were studied. Multi-criteria plan optimisation based on a set of “wish-list” prescription objectives, together with the selection of beam geometries from libraries of candidate directions enabled the development of sets of Pareto-optimal IMRT plans. In 97 % of the cases tested in a prospective clinical study for head and neck cancer, the automatically generated plans resulted preferable [27, 28].

Among these, methods to automatically adjust the objectives during optimisation (autoplan concept) enabled some degree of automation [29, 30]. Also data-driven methods were developed in research-based planning systems. The investigation of the spatial relationships between OARs and targets and the automatic generation of objectives derived from a database of cases and used as initial planning goals was demonstrated to be reliable and suggestive of possible automation of IMRT planning [31–33].

Knowledge-based planning methods (or Pareto-optimal multi-criteria methods) do not eliminate the need to perform individual plan optimisation and calculation. The scope of these approaches is to automate and individualise at maximum the process of optimisation with the definition of the best constraints possibly minimizing interactive and iterative interventions. The actual optimisation and final calculation can already today be made automatic by the existing commercial implementations so, with the demonstration of the reliability of knowledge based methods, a further step towards complete automation of the inverse planning process is achieved.

It is of course true that the findings presented in this study are depending upon the specific software solution implemented by the vendor and are directly applicable only to centres having similar infrastructures. Nevertheless, the role of knowledge-based optimisation engines and the clinical usability of these methods has a more general value and might give insights to future directions of developments of the radiotherapy chain.

In the present study, the case of esophageal cancer was chosen to investigate the performance of a broad-scope knowledge-based model. The concept of broad-scope was realised with, on one side, the selection of a heterogeneous set of plans for the training (wide range of dose prescriptions, huge variation in target size and localisation from the upper to the lower third - with partial inclusion of the stomach -) to predict patient tailored dose-volume objectives for inverse planning. The relevant variation in dose prescription was due to the different localisation of the target, the various trade-off with OARs, and the different protocols applied. The variation in target size and shape (from short-symmetric targets in the upper third to very elongated-symmetrical in the central and central-lower to the elongated and asymmetric-lateral targets of the lower third) added complexity to the optimisation process. The presence of many OARs, with very different sizes and geometrical relationships to the target induced several trade-off problems and challenged the achievement of the planning objectives. The sub-analysis on two groups with symmetric or asymmetric targets did not lead to any significant difference compared to the main results. This was expected since the model was trained with a cohort of mixed cases with equivalent incidence of both classes. The DVH estimation engine demonstrated a sufficient generalisation power and generated adequate predictions for both groups during the validation. Similarly no difference was found in diviging the group of patients by upper, medium or lower third of the esophagus for the same reason. A training set which samples the patient population with an adequate case mix can be used for a general purpose. .

As a second side of the broad-scope investigation, the model validation was performed on three groups of cases (not used for the training). Two sets of cases from each of the two clinics contributing to the training and one set from a third, external, clinic. This was similar to the concept investigated by Good et al. in their study [5]. Only if the model-based approach gives results equivalent or superior to the corresponding reference plans optimised with traditional methods in the external clinic, the validity of the model and its broad-scope are proven. The here presented results demonstrated that this was indeed the case, and the model, built with 70 patients from clinics A and B resulted adequate to properly optimise plans from clinic C. The actual exchange of the model between centers would be quite easy since all the necessary data (the parameters fit from the training phases) can be exported in binary encrypted format from one center and simply reimported into the Eclipse planning system of the destination center. No exchange of any patient data would be necessary for the purpose.

From an operational perspective, the RapidPlan knowledge-based engine allowed to generate and train a predictive model of clinically acceptable performance. The number of cases used for training was 70, greater than what used in the liver or lung and prostate experiments (27 or 45) or in the head and neck study (30 or 60), but still reasonably limited to allow patient selection in a medium sized clinic. The training set was determined without special selection criteria. The guideline followed for the study was to include in the training set an adequate representation of the population to be sampled. In the present case, three main subgroups were identified according to the location of the tumor and the number of training cases used scaled with the number of classes times the minimum number of patents per OAR to build a model (which is 20). The results obtained thus reinforce the possibility to build effective broad-scope models and, likely, suggest that, to some extent, the use of heterogeneous datasets (in their geometric and dosimetric aspects) might be useful if not necessary. Further studies are needed to determine the correlation between heterogeneity of the input data, number of training cases needed, and generalisation power of the models. The present results suggest that modest numbers are sufficient to represent wide clinical conditions.

The detailed analysis of the residual planning criteria violations, showed that the knowledge-based plans presented fewer failures than the original clinical plans but still not all criteria were met for all patients (the 3 cases of class 2 pass-fail findings). The present results are generated with no user interaction during the optimisation runs, and therefore might suggest some challenge when complex trade-offs are present. In such cases it would be possible to apply manual interactive refinements during the optimisation.

The validation based on patients originated from a department not member of the training group, as in the present study, is the proof of the broad-scope model concept value. The fact that with RapidPlan it was possible to generate plans systematically equivalent or superior to the reference also for this centre, demonstrates a good power of generalisation. As a consequence, the transfer of the planning knowledge from more experienced and profiled centres to either less advanced or more busy institutions is shown to be possible. It would possibly be conceivable the use of properly built broad-scope models to harmonise and uniform the planning phases for multicentric trials (provided compatible systems are available).

## Conclusions

RapidPlan, a novel knowledge-based DVH estimation model was successfully configured, trained and validated for esophageal cancer. The system allowed to improve the quality of VMAT RapidArc plans against the reference clinically accepted plans. In particular RapidPlan based plans resulted superior to reference plans for the cases provided by a clinical centre not having contributed to any case in the model training phase. Results are suggestive for a reliable application of the methodology to the clinical routine.

## Additional file

Additional file 1: Supplementary materials further describing the Rapidplan logic and complementing the numerical results summarised in the main text. (DOCX 2378 kb)
