# Supplementary material for: A broad scope knowledge based model for optimization of VMAT in esophageal cancer: validation and assessment of plan quality among different treatment centers
Source: Radiat Oncol. 2015 Oct 31;10:220. doi: 10.1186/s13014-015-0530-5 (PMC4628288; doi:10.1186/s13014-015-0530-5)

**A broad scope knowledge based model for optimization of VMAT in esophageal cancer. Validation and assessment of plan quality among different treatment centers.**

**Supplementary materials**

A new optimization engine was introduced in the Eclipse TPS (Varian Medical Systems, Palo Alto, USA) in the release 13.5. This is made of three main components: a model building and training engine; a model-based dose-volume histogram (DVH) and automated constraints prediction tool; a new VMAT and IMRT optimization algorithm to manage the above.

The sequence of main steps necessary to generate a model is as follows:

- Selection of a set of training plans. No specific requirements are mandatory to be candidate for training. The strategy adopted was to create a “universal” model for HCC and the only requirement was to select plans accepted for clinical treatment.
- Association of these plans to a model layout where target and organs at risk ontology, dose prescriptions and some descriptive elements can be defined.
- Definition of the type of constraints to be generated per each structure (points vs. lines, priorities, user defined vs. fully automated).
- Dosimetric and geometrical data extraction from the patient database to the model engine.
- Model training. Based on principal component (PC) analysis methods [2,3,15,16].
- Model publication and validation.

Figure 1 shows a schematic representation of the model determination steps and of the PC method applied to DVH. The assumption is that any DVH can be represented as a combination of the average DVH over a population plus a sum of some weighted PC and a residual. The first PC is determined by maximizing the variance of the training set it can explain; any consecutive PC is chosen so that the residual variance is further accounted for. The features used to build the model primarily include geometric characteristic of the various structures, as well as their mutual position and their relationships with the treatment fields. These are modeled by constructing a Geometry-Based Expected Dose (GED) which evaluates the distance between each structure and the target surface by means of the amount of dose that each target contributes to an organ for the current field geometry. The final prediction model is built as a combination of the PC and regression techniques for the in-field region of any OAR and a mean and standard deviation model on the DVH fo the other OAR regions. The PC is applied to the GED and DVH to find the main component scores.

A trained model, once made “public”, can be used to perform predictive estimation of the DVHs for any given new test case and, from these, to determine the planning constraints. The DVH prediction workflow is described as:

- Selection of a knowledge-based model
- Matching of structure names if the ontology mapping is not complete
- Prediction of a range of possible DVH for each of the structures present in both the plan and the model.
- Automatic generation of the dose-volume constraints based on the rules from the model configuration. With a fully automatic procedure, these are located below the lower limit of a prediction range generated from the most probable DVH curve by adding and subtracting a variation curve. This corresponds to 1 standard deviation for the out-of-field region. For the in-field region this is constructed by adding in quadrature the DVH PC multiplied by the standard error related to the model regression. Also the priorities are defined by the prediction engine and account for a basic balance between all possible trade-offs. All point constraints and priorities can be modified during optimization.

**Figure S1:** The schematics and the logic at the base of the RapidPlan implementation


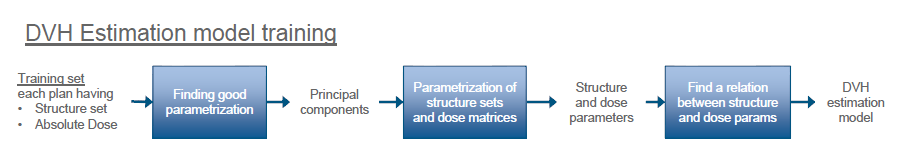


DVH estimation

Estimated DVHs

Automatic objective generation

Patient information

Structure sets

Prescription

Field geometry

Optimization objectives

Optimize

DVH Estimation Algorithm


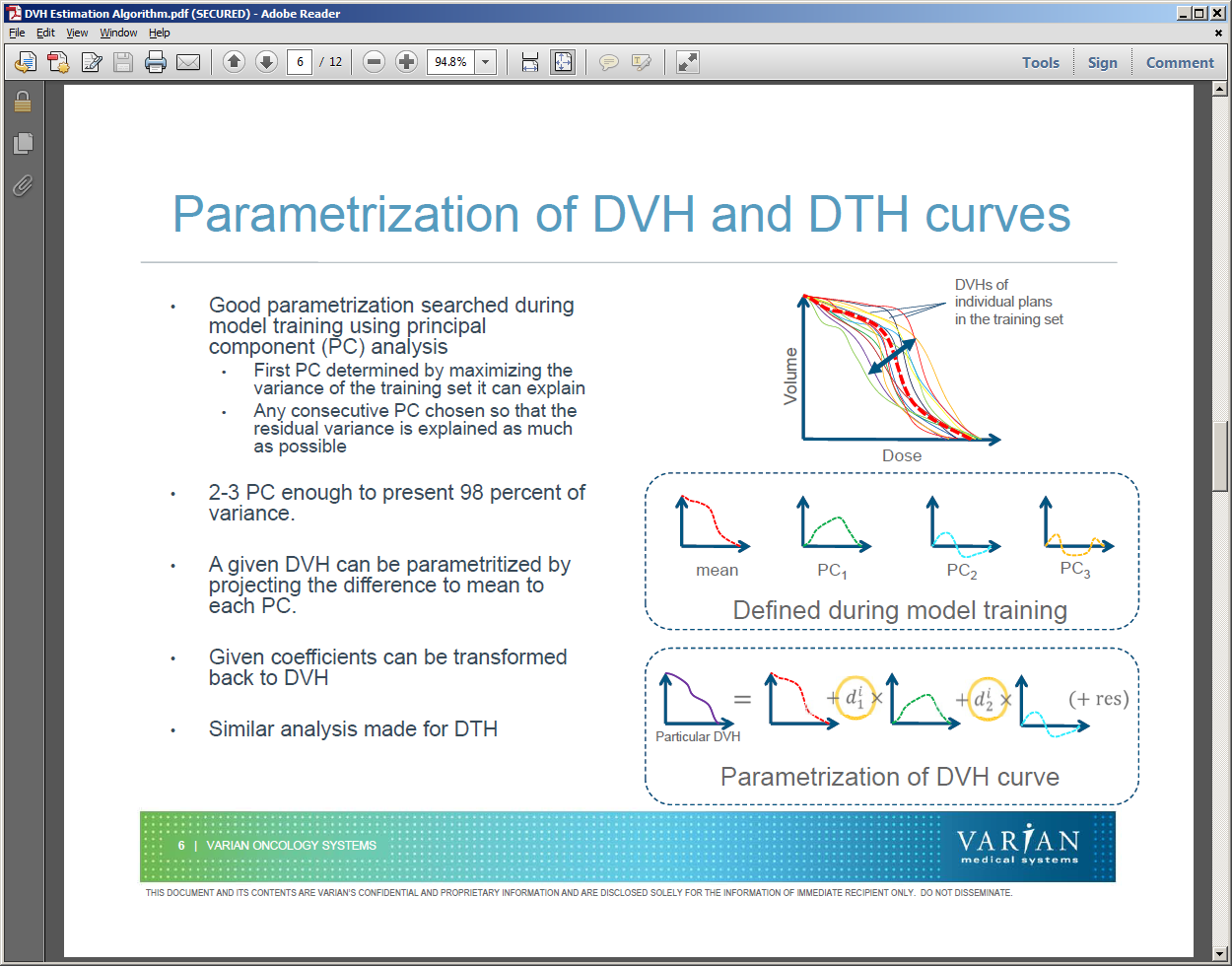


Principal components schematics


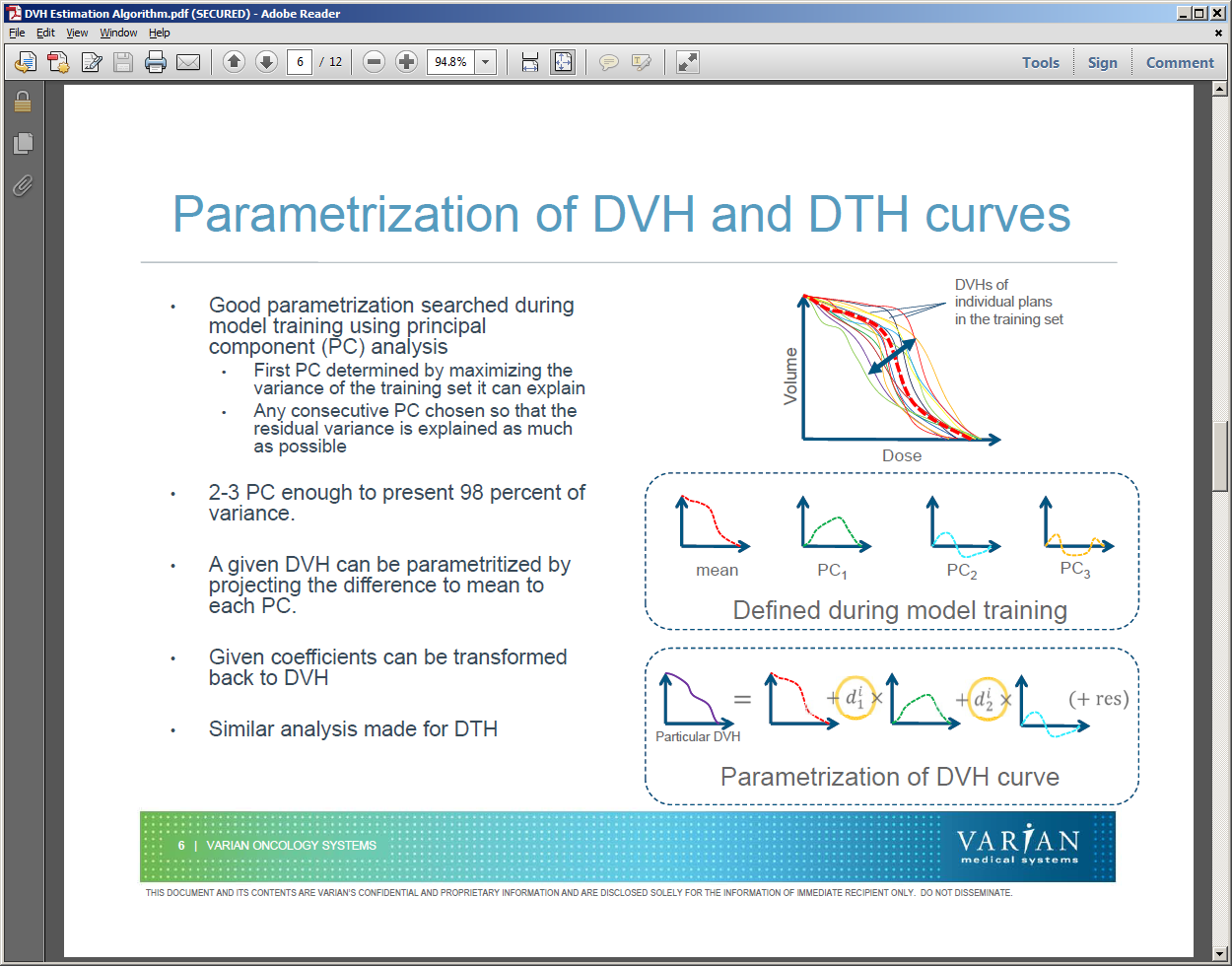


**Table S1:** dose-volume constraints structure in the RadpiPlan model.

| Structure | Objective type | Relative volume (%) | Dose (% of prescription or absolute dose in Gy) | Priority |
| --- | --- | --- | --- | --- |
| CTV | Upper  Lower | 0  100 | 101%  100% | Generated  Generated |
| PTV | Upper  Lower | 0  100 | 101%  100% | Generated  Generated |
| Lung left | Mean  Line | Generated | Generated  Generated | Generated  Generated |
| Lung right | Mean  Line | Generated | Generated  Generated | Generated  Generated |
| Heart | Line | Generated | Generated | Generated |
| Trachea | Line | Generated | Generated | Generated |
| Spinal cord | Line | Generated | Generated | Generated |
| Stomach | Line | Generated | Generated | Generated |
| Bowel | Line | Generated | Generated | Generated |
| Kidney left | Line | Generated | Generated | Generated |
| Kidney right | Line | Generated | Generated | Generated |
| Liver | Line | Generated | Generated | Generated |
| Spleen | Line | Generated | Generated | Generated |

**Table S2:** Summary the DVH analysis for the reference and the RapidPlan plans for the cohort of validation patients from the clinic A.

|  | **Objective** | **Reference** | **RapidPlan** | **p** |
| --- | --- | --- | --- | --- |
| **PTV** | | | | |
| Mean [%] | 100% | 100.0±0.0 | 100.0±0.0 | - |
| D_1%_ [%] | <107% | 104.5±4.2 | 104.0±3.5 | - |
| V_95%_ [%] | >95% | 97.6±3.5 | 98.2±1.4 | - |
| St. Dev. [Gy] | <5% | 2.0±0.1 | 1.8±0.1 | - |
| **Left lung** | | | | |
| Mean [Gy] | <15Gy | 7.2±3.9 | 6.7±3.5 | 0.03 |
| V_20Gy_ [%] | <20% | 9.0±7.8 | 7.5±6.77 | - |
| **Right lung** | | | | |
| Mean [Gy] | <15Gy | 7.0±3.5 | 6.4±3.2 | 0.09 |
| V_20Gy_ [%] | <20% | 6.4±5.8 | 4.9±4.1 | 0.09 |
| **Heart** | | | | |
| Mean [Gy] | minimise | 9.3±6.5 | 9.4±6.6 | - |
| V_30Gy_ [%] | <30% | 4.1±5.1 | 4.3±5.4 | - |
| **Spine** | | | | |
| D_1cm3_ [Gy] | <45Gy | 21.1±5.4 | 18.8±5.6 | 0.03 |
| **Liver** | | | | |
| Mean | minimise | 8.9±4.3 | 8.3±4.1 | - |
| **Spleen** | | | | |
| Mean [Gy] | minimise | 11.6±6.7 | 9.5±5.6 | 0.06 |
| D_1%_ [Gy] | minimise | 29.0±10.9 | 26.2±11.4 | 0.06 |
| **Left kidney** | | | | |
| V_15Gy_ [%] | <35% | 2.9±3.3 | 0.8±1.3 | - |
| **Right kidney** | | | | |
| V_15Gy_ [%] | <35% | 0.2±0.3 | 1.3±2.3 | - |
| **Stomach** | | | | |
| D_1%_ [Gy] | minimise | 40.4±7.6 | 38.8±9.3 | - |
| **Small bowel** | | | | |
| Mean [Gy] | minimise | 5.1±2.3 | 4.7±2.2 | - |
| D_1%_ [Gy] | <45Gy | 18.8±4.5 | 18.6±4.1 | - |

**Table S3:** Summary the DVH analysis for the reference and the RapidPlan plans for the cohort of validation patients from the clinic B.

|  | **Objective** | **Reference** | **RapidPlan** | **p** |
| --- | --- | --- | --- | --- |
| **PTV** | | | | |
| Mean [%] | 100% | 100.0±0.0 | 100.0±0.0 |  |
| D_1%_ [%] | <107% | 103.8±0.9 | 104.0±08 | - |
| V_95%_ [%] | >95% | 97.5±2.2 | 98.2±0.9 | - |
| St. Dev. [Gy] | <5% | 1.9±0.3 | 1.8±0.2 | - |
| **Left lung** | | | | |
| Mean [Gy] | <15Gy | 9.1±3.1 | 9.0±3.3 | - |
| V_20Gy_ [%] | <20% | 7.1±4.2 | 7.4±3.9 | - |
| **Right lung** | | | | |
| Mean [Gy] | <15Gy | 9.1±4.0 | 8.7±3.5 | - |
| V_20Gy_ [%] | <20% | 8.2±6.2 | 8.0±4.7 | - |
| **Heart** | | | | |
| Mean [Gy] | minimise | 12.6±7.2 | 11.6±6.8 | 0.03 |
| V_30Gy_ [%] | <30% | 7.5±5.5 | 7.2±5.2 | - |
| **Spine** | | | | |
| D_1cm3_ [Gy] | <45Gy | 18.8±6.0 | 18.2±5.3 | - |
| **Liver** | | | | |
| Mean | minimise | 12.2±4.8 | 11.7±4.7 | - |
| **Spleen** | | | | |
| Mean [Gy] | minimise | 19.7±7.6 | 16.5±6.1 | 0.06 |
| D_1%_ [Gy] | minimise | 38.8±5.6 | 38.4±6.1 | - |
| **Left kidney** | | | | |
| V_15Gy_ [%] | <35% | 28.2±17.1 | 24.0±25.3 | - |
| **Right kidney** | | | | |
| V_15Gy_ [%] | <35% | 8.7±10.1 | 3.8±5.9 | - |
| **Stomach** | | | | |
| D_1%_ [Gy] | minimise | 42.2±0.4 | 42.6±0.3 | 0.03 |
| **Small bowel** | | | | |
| Mean [Gy] | minimise | 8.6±3.4 | 7.7±3.1 | 0.06 |
| D_1%_ [Gy] | <45Gy | 32.3±5.9 | 31.0±7.6 | - |

**Figure S2:** The distribution of DVH used for the model configuration and training.

**
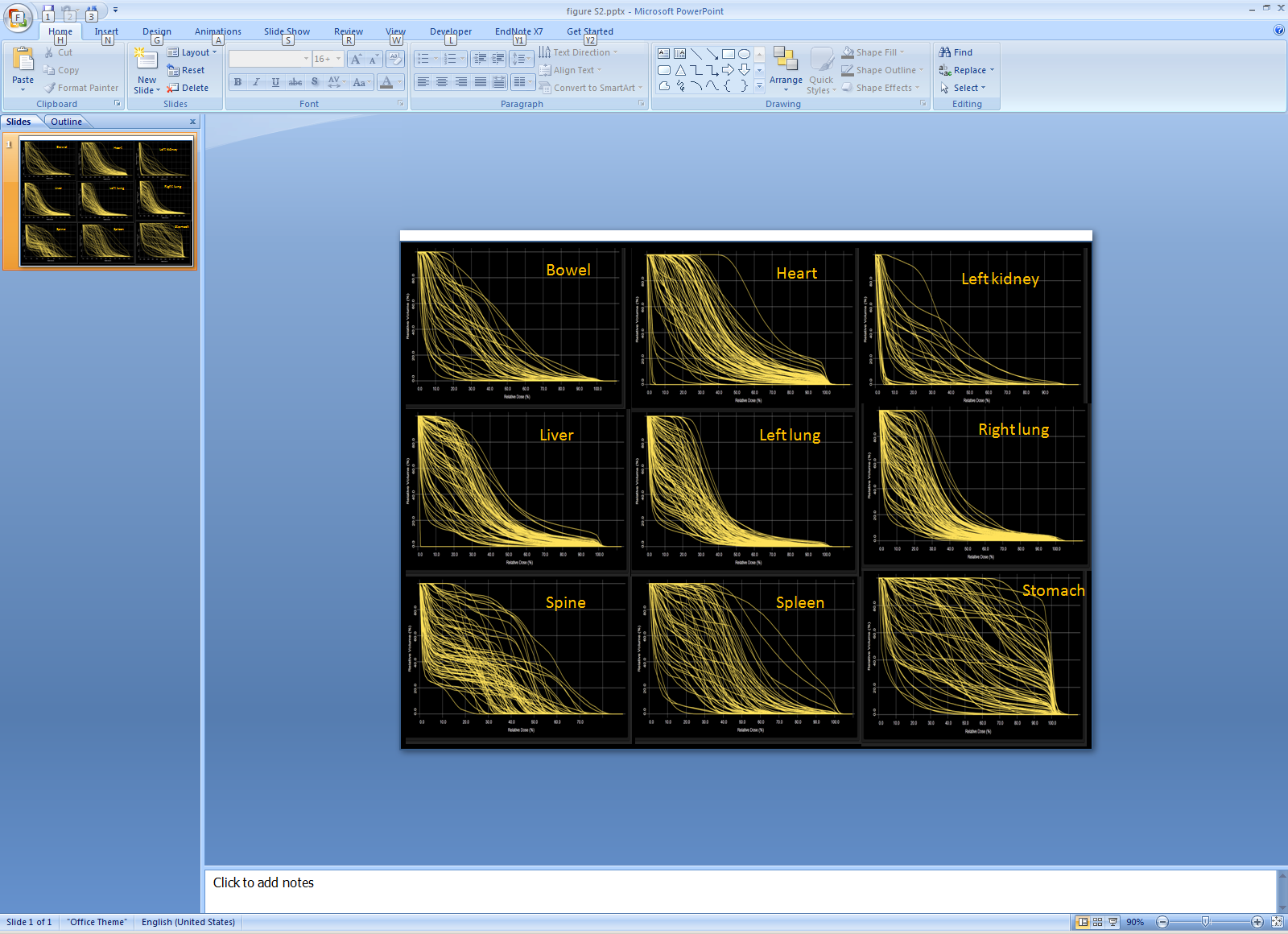
**

**Figure S3:** Scatter plot and regression fit lines for the residual estimates from the model training phase for some of the organs at risk.


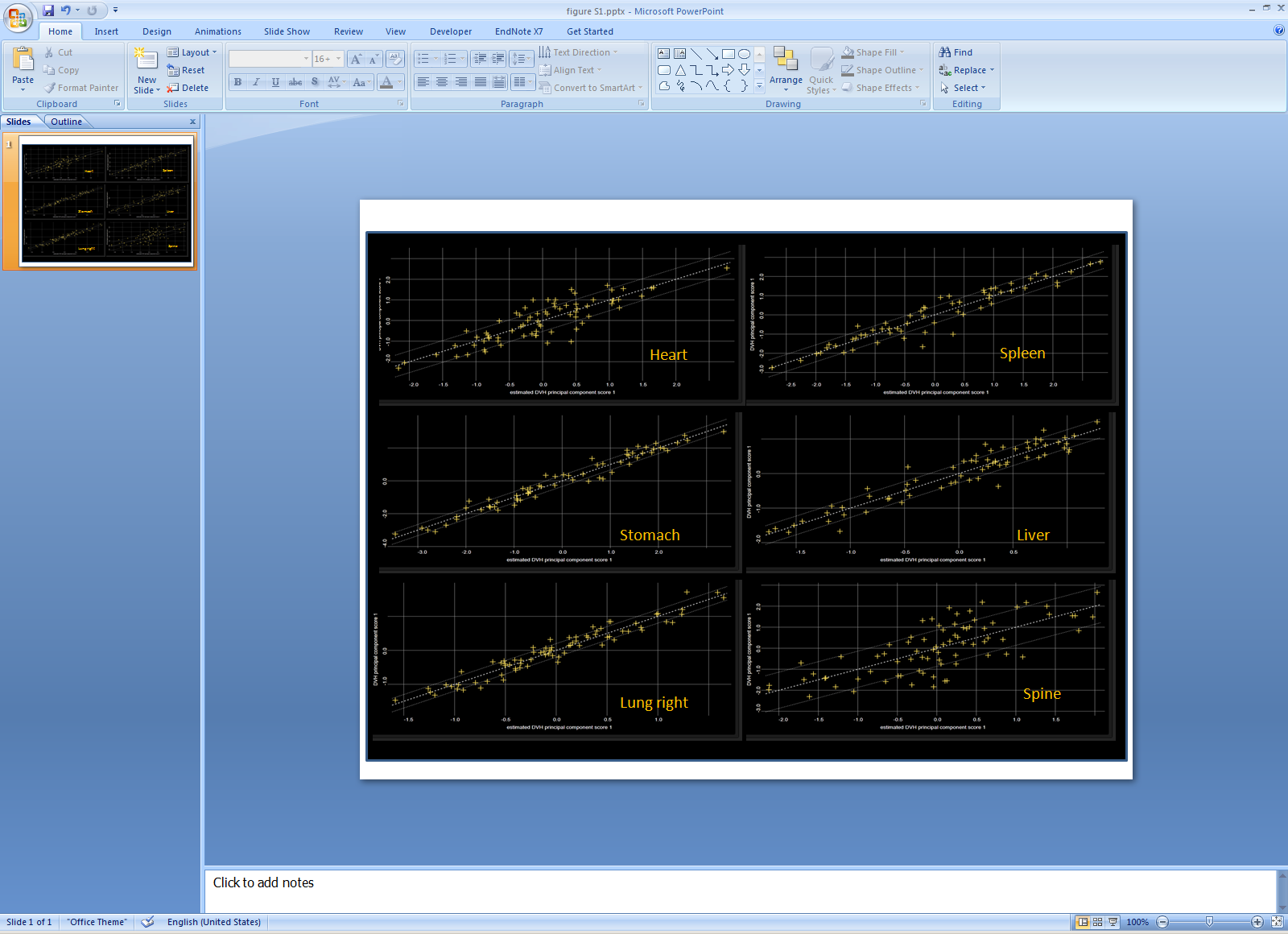


**Figure S4**: The dose volume histograms for the validation experiment of the clinic A


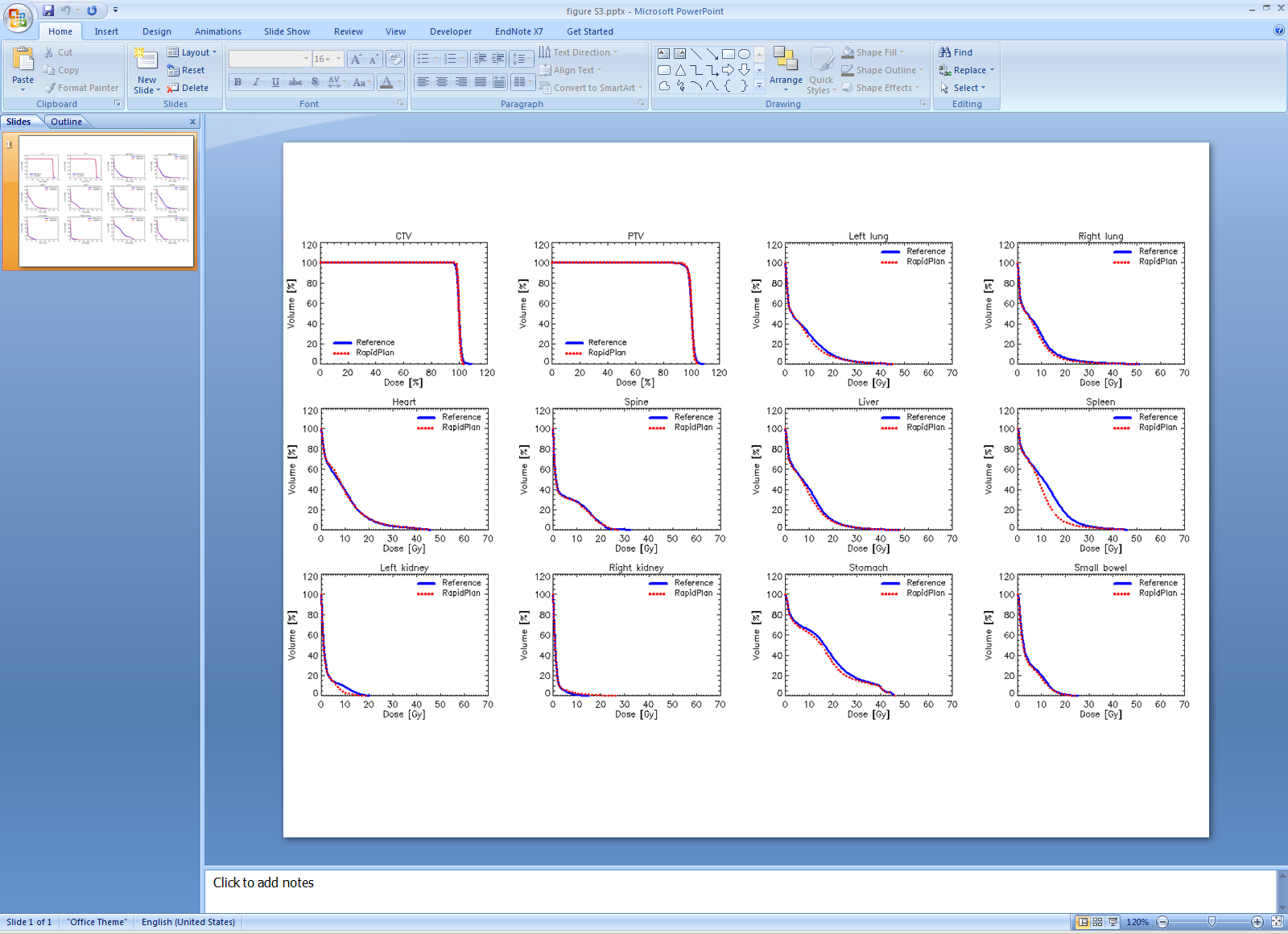


**Figure S5:** The dose volume histograms for the validation experiment of the clinic B


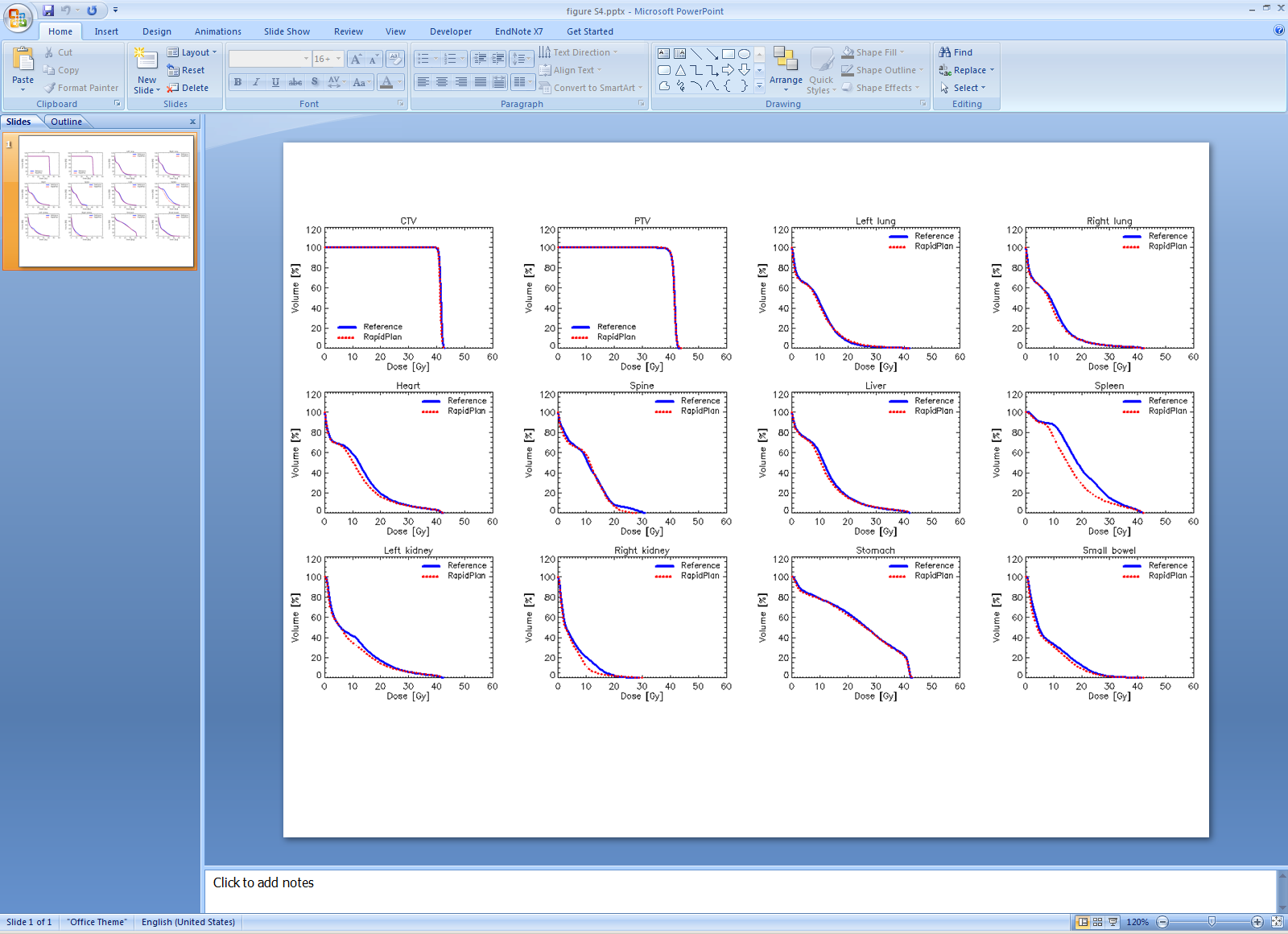


**Figure S6:** Average DVH for the OARs in the subanalysis performed on the groups of patients with rotationally symmetric or asymmetric targets. Despite the differences in the dose levels, the general features observed for the global cohort were maintained. This implies that the model, built on a mix of cases from the two subgroups, is capable to manage all geometrical conditions.


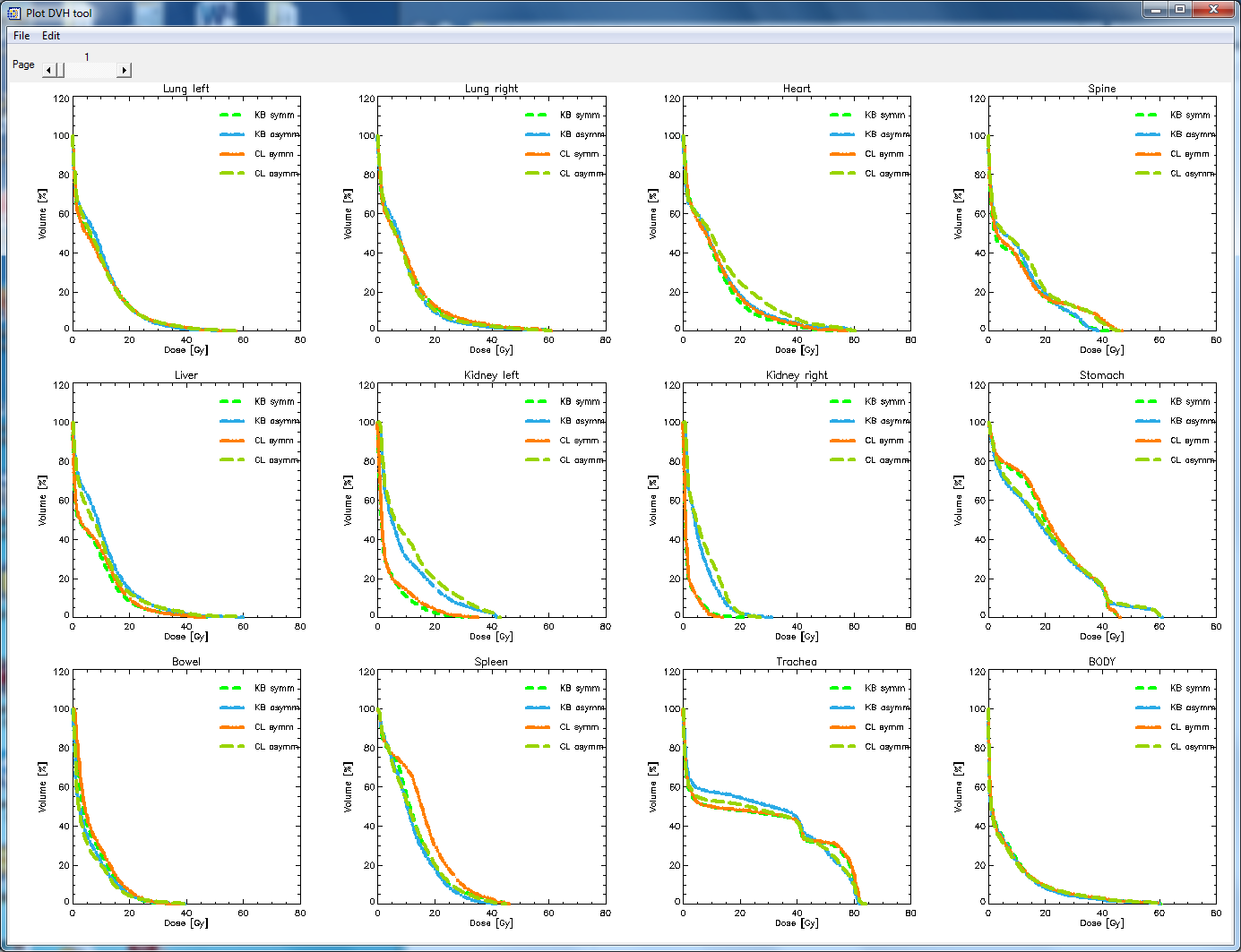

Supplement: Supplementary file 1 — Supplementary materials further describing the Rapidplan logic and complementing the numerical results summarised in the main text. (DOCX 2378 kb) [file 13014_2015_530_MOESM1_ESM.docx]
